# A Survey of Enhanced Recovery After Surgery Protocols for Cesarean Delivery in Serbia

**DOI:** 10.3389/fmed.2018.00100

**Published:** 2018-04-17

**Authors:** Borislava Pujic, Mirjana Kendrisic, Matthew Shotwell, Yaping Shi, Curtis L. Baysinger

**Affiliations:** ^1^Clinical Center Vojvodina, Obstetrics and Gynecology Hospital, Novi Sad, Serbia; ^2^General Hospital, Sremska Mitrovica, Serbia; ^3^Department of Biostatistics, School of Medicine, Vanderbilt University, Nashville, TN, United States; ^4^Department of Anesthesiology, School of Medicine, Vanderbilt University, Nashville, TN, United States

**Keywords:** cesarean delivery, enhanced recovery, neuraxial anesthesia, length of stay, obstetric anesthesiology

## Abstract

Enhanced recovery after surgery (ERAS) protocols have been described for patients undergoing colon surgery. Similar protocols for cesarean delivery (CD) have been developed recently. CD is one of the most commonly performed surgical procedures, and adoption of ERAS protocols following CD might benefit patients and the health-care system. We aimed to determine which Serbian hospitals reported ERAS protocols, which elements of ERAS protocols were used in CD patients, and whether ERAS and non-ERAS hospitals differed. The survey was sent to all hospitals with obstetric services and 46 of 49 responded. The questionnaire asked whether ERAS protocols had been formally adopted for surgical patients and about their use in CD patients. Specific questions on elements described in other obstetric ERAS protocols for CD included preoperative patient preparation, type of anesthesia and temperature monitoring used for CD, maternal/neonatal contact, and time to discharge. ERAS protocols are used in 24% of surveyed hospitals, 84% admit the patient the day before elective CDs, 87% use a maternal bowel preparation morning on the day of CD, and 80% administer maternal deep venous thrombosis prophylaxis. Only 33% remove IV in the first postoperative day, and 89% of women do not eat solid food until the day following their CD. Neuraxial anesthesia is used in 46% of elective CDs in ERAS hospitals compared to 9% in non-ERAS hospitals (*P* < 0.01), and neuraxial narcotics for post CD analgesia are given more often in ERAS hospitals. Thirty-six percentage of ERAS patients are discharged within 3 days vs. none in the non-ERAS group. Few elements of ERAS protocols reported from other centers outside Serbia are employed in Serbian hospitals performing CD. Despite significant changes that have been made recently in CD care, enhanced recovery after CD could be significantly improved in Serbian hospitals.

## Introduction

The significance of enhanced recovery after surgery (ERAS) protocols has been well established in non-obstetric surgery patients and was described by Wilmore and Kehlet, 15 years ago (1). Soon after ERAS protocol successes in speeding patient recovery, decreasing times to discharge form the hospital and improving patient outcomes were described in colorectal surgery patients (2), successful application was reported in urological, breast, pancreatectomy, liver resection, and gynecologic surgery (3–9). The ERAS approach emphasizes optimization of the processes of patient care, so that enhanced patient recovery can occur without decreasing the quality of care or patient satisfaction. ERAS aims to return the patient to normal life quickly. The increased efficiencies have been demonstrated to reduce waste of scarce resources (10) and thus reduce the overall cost of care. While significant variations in ERAS protocols exist both within and between surgical specialties (11), common elements often include effective patient education and acceptance, good perioperative hydration and nutrition, use of surgical techniques associated with fastest patient recovery, maintenance of perioperative patient normothermia, early removal of urinary catheters, adequate pain relief that promotes early ambulation and minimal use of perioperative opioids (which helps the return of bowel function quickly). The multidisciplinary approach requires commitment from the patient, as well as all of the persons involved in their perioperative care: surgeons, anesthesiologists, pain specialists, nursing staff, physical and occupational therapists, social services, and hospital administration. Successful ERAS protocol implementation involves the creation of a core team of anesthesiologists, obstetricians, specialist nurses, and hospital managers (12). The large number of elements that are required for implementation of an ERAS protocol often requires significant culture change in the health organization (12).

### Background and Rationale

Enhanced recovery after surgery protocols have been developed for patients undergoing cesarean delivery (CD) in recent years. CD is one of the most commonly performed operations worldwide, its incidence is increasing, and elective CD accounts for an increasing proportion of those operations (13). Therefore, CD represents an increasing burden on national health-care systems. Recent guidelines of the UK National Institute for Health and Care Excellence suggest that a majority of women undergoing elective CD could be discharged on the day after delivery (14). Such early discharges are not associated with either increased patient morbidity or higher readmission rates than in women discharged later (15). One recent report suggested that many women would prefer to be discharged a day earlier than occurred following their delivery (16). The widespread adoption of ERAS protocols for CD was recently advocated by Lucas and Gough as a means for achieving these positive outcomes (17).

Reports describing the implementation of ERAS protocols for CD have been recently published (13, 16, 18–21). In addition to the ERAS elements used in non-obstetric surgeries, these reports often describe early skin-to-skin contact in theater between mother and neonate, infant temperature monitoring to prevent neonatal hypothermia, delayed cord clamping, and early breastfeeding after maternal breast-feeding education. Most of these reports are from the UK, and two survey reports from there note a growing consensus of the elements that ERAS for CD should contain (21, 22).

No reports describing ERAS protocols for CD from Eastern European or middle-income countries have been published, and the number of institutions that use them are unknown. Accordingly, we aimed to determine the use of ERAS protocols for CD in Serbia and to determine the differences in use of common ERAS elements between those institutions that report the use of ERAS protocols and those that do not.

## Materials and Methods

A survey tool of 22 questions with multiple choice answers was sent by email to all hospitals in Serbia (4 university teaching hospitals and 45 general hospitals), which provide obstetric services (Table 1). This survey was approved by Ethical Committee at the Clinical Center of Vojvodina. The questionnaire was completed by either the Chief of Obstetrics or Chief of Anesthesiology who had knowledge of all aspects of perioperative care within the institution, and only one questionnaire per institution was returned. One of the authors (BP) followed up after distribution of the survey to answer all responder questions. The questionnaire asked for the presence of ERAS protocols for CD in the hospital, preoperative patient counseling of the elements within the protocol, time of admission to hospital and hospital stay, bowel preparation prior surgery, antibiotic prophylaxis prior to skin incision, prevention of deep venous thrombosis (DVT), percentage of neuraxial anesthesia (NA) for elective and emergency CD, use of intrathecal analgesia, medications for postoperative analgesia, oral intake after CD, use of chewing gum in the early postoperative period, duration of IV fluid therapy, urinary catheter removal, early mobilization of the parturient, skin-to-skin contact between mother and baby in the operating theater, monitoring of body temperature, and active maternal warming intraoperatively. Overall responses were recorded, and the responses between those hospitals with ERAS protocols in place were compared with those who did not report having them. Pearson’ chi square test was used where appropriate for comparisons between groups in this prospective observational study (R version 3.3.3, R Core Team, R Foundation for Statistical Computing). Differences of *P* ≤ 0.05 were considered significant.

**Table 1:** Questionnaire.

INSTITUTION _______________________
SPECIALTY (around)
– Obstetrics – Anesthesiology

**QUESTIONAIRE**
In this research are included all hospitals in Serbia (4 university and 45 general Hospitals). Please answer the following questions by around the letter in front of the right answer or you write your answer. Filled questionnaire return by e-mail to following addresses:
borislava60@yahoo.com
mkendrisic@yahoo.co.uk
According to your answers, we shall try to understand which of the “Fast track” surgery criteria (ERAS: enhanced recovery after surgery) are implemented in our hospitals and to make some guidelines for cesarean delivery (CD).

Is the ERAS protocol for better and faster recovery after cesarean section introduced to the parturients in your institution? Yes No Sometimes __________________

2. Who inform the parturients preoperative? Obstetrician who takes care during the pregnancy Obstetrician who perform CS Anesthesiologist in anesthesia clinic Anesthesiologist who perform anesthesia for CS _____________________________

3. In your hospital is usual patient’s admission prior CS? Evening before schedule surgery 24 h before In the morning, on the surgery day ________________________________

4. In your institution do you use bowel preparation before CS? Yes No Sometimes __________________

5. In your institution do you use antibiotics prophylaxis 30’ before CS? Always Sometimes Never __________________

6. In your institution do you use DVT prophylaxis before or after CS? Always Sometimes Never __________________

7. What is the percentage of regional anesthesia (RA) for scheduled CS in your institution? <10% 10–29% 30–49% >50%

8. What is the percentage of RA for emergent CS in your institution? <10% 10–29% 30–49% >50%

9. Do you use opiates intrathecal for postoperative analgesia (morphine)? Always Sometimes Never __________________

10. Analgesia following CS is: IV IM combination (IV and IM) per oral __________________

11. How long parturients are on the IV infusion? < 24 h 24 h 48 h Depends of case __________________

12. When do you start with per oral liquids intake? Immediately postoperative Following 12 h Following 24 h Following 48 h

13. When do you start with per oral food intake? Following 12 h Following 24 h Following 48 h __________________

14. Do you use chewing gum following CS? Yes No Sometimes

15. When they start walking after CS? In the evening (On the day of CS) Tomorrow morning (following 24 h) Following 48 h __________________

16. When do you remove urinary catheter following CS? On the day of CS Tomorrow Following 48 h Depends of case

17. Do you use “skin to skin” contact on the operating table? Yes No

18. Do you use temperature checking intraoperative? Yes No

19. Do you use active warming during CS? Yes No Sometimes

20. Do you use medication to prevent chronic pain (gabapentin or pregabalin)? Yes No __________________

21. How many CS do you have at your hospital per year? <500 501–1,000 >1,000

22. How long are parturients at the hospital following CS before discharge home? <3 days 4–6 days >6 days

## Results

Responses were obtained from 46 of 49 hospitals (3 university and 43 general hospitals; a 94% response rate). ERAS protocols were in use in 11 of 46 (24%) of surveyed hospitals and 63% of the time the responsibility for patients counseling was shared between the obstetrician and anesthesiologist (Table 2).

**Table 2:** Survey items with similar responses from institutions with and without ERAS protocols.

Item	Overall	ERAS protocol	No ERAS protocol	*P* value
1. ERAS protocol is used	11 (24%)			
2. Who educates patient?				0.47
Obstetrician	9 (20%)	1 (9.1%)	8 (23%)	
Anesthesiologist	8 (17%)	3 (27%)	5 (14%)	
Either	29 (63%)	7 (64%)	22 (63%)	
3. When admitted for CD				0.17
Day before	37 (84%)	11 (100%)	26 (79%)	
Day of	7 (16%)	0	7 (21%)	
4. Bowel prep is used				0.35
Yes	39 (87%)	11 (100%)	28 (82%)	
No	4 (9%)	0	4 (9%)	
Sometimes	2 (4%)	0	2 (4%)	
5. Antibiotics 30 min before CD				0.08
Yes	21 (51%)	8 (73%)	13 (43%)	
No	5 (12%)	2 (18%)	3 (10%)	
Sometimes	15 (37%)	1 (9%)	14 (47%)	
6. DVT prophylaxis				0.29
Yes	37 (80%)	11 (100%)	26 (74%)	
No	1 (2%)	0	1 (3%)	
Sometimes	8 (17%)	0	8 (23%)	
7. Neuraxial narcotics for CD				0.09
Yes	4 (9%)	2 (18%)	2 (6%)	
No	25 (56%)	3 (27%)	22 (65%)	
Sometimes	16 (35%)	6 (55%)	10 (30%)	
8. Parenteral narcotics administration				0.56
IV	14 (36%)	2 (22%)	12 (40%)	
IM	1 (3%)	0	1 (3%)	
Both	24 (61%)	7 (78%)	17 (57%)	
9. When IV is removed				0.16
Immediately after CD	0	0	0	
<24 h after CD	13 (33%)	6 (55%)	7 (24%)	
24–48 h after CD	26 (65%)	5 (45%)	21 (72%)	
>48 h after CD	1 (2.5%)	0	1 (3%)	
10. When solid food is allowed				0.89
Immediately after CD	0	0	0	
12 h after CD	5 (11%)	1 (9%)	4 (12%)	
24 h after CD	20 (44%)	6 (55%)	14 (41%)	
48 h after CD	20 (44%)	4 (36%)	16 (47%)	
11. Chewing gum is used				0.15
Yes	0	0	0	
No	36 (86%)	10 (100%)	26 (74%)	
Sometimes	9 (20%)	0	9 (26%)	
12. Urinary catheter removed				0.44
Day of CD	2 (5%)	0	2 (6%)	
First post-operative day	26 (59%)	8 (73%)	18 (55%)	
Second post-operative day	11 (25%)	3 (27%)	8 (24%)	
Clinician judgment	5 (11%)	0	5 (15%)	
13. Skin to skin contact				0.09
Yes	22 (49%)	8 (73%)	14 (41%)	
No	23 (51%)	3 (27%)	20 (59%)	
14. Monitor maternal temp				0.32
Yes	5 (11%)	0	5 (14%)	
No	41 (89%)	11 (100%)	30 (86%)	
15. Active warming during CD				0.24
Yes	2 (4%)	0	2 (6%)	
No	34 (76%)	7 (64%)	27 (80%)	
Sometimes	9 (20%)	4 (36%)	5 (14%)	
16. Routinely give gabapentin				1.0
Yes	10 (22%)	2 (18%)	8 (23%)	
No	36 (78%)	9 (82%)	27 (77%)	

*P values calculated using the Pearson Chi square test*.

*CD, cesarean delivery; NA, neuraxial anesthesia; h, hours; temp, temperature; admin, administration; DVT, deep venous thrombosis; min, minutes; ERAS, enhanced recovery after surgery*.

### Survey Elements With Similar Responses Between ERAS and Non-ERAS Hospitals

Many surveyed items did not vary between institutions that reported ERAS use vs. those that did not. Surveyed hospitals reported admitting the patient the day before elective CD 84% of the time, use of maternal bowel preparation in the morning on the day of CD in 87% of patients, measures to prevent maternal DVT during the peri-partum period in 87% of mothers, administration of gabapentin approximately 22% of the time as an analgesic adjunct, with no differences between ERAS and non-ERAS groups.

Perioperative maternal temperature monitoring was reportedly used only 11% of the time and active warming of either IV fluids or other active measures was used less than 5% of the time; there were no differences between ERAS and non-ERAS groups. The maternal urinary catheter was nearly always retained until at least the first postoperative day and was removed in approximately a quarter of women on the second day, with removal in the rest after that. Only 5% of women had their IV removed within 24 h after delivery and 88% did not have solid food until the day following their CD. Among hospitals reporting ERAS use, 73% administered antibiotics within 30 min of a CD, compared to 43% in non-ERAS hospitals, and skin-to-skin contact during CD under NA occurred in 73% of ERAS institutions compared to less than 43% of non-ERAS hospitals, differences that nearly reach statistical significance. The use of neuraxial narcotics for postoperative analgesia is rare in Serbian hospitals, used 9% of the time overall, but 18% of ERAS protocol hospital reported using them vs. 6% of non-ERAS institutions, a difference that also nearly reached statistical significance.

### Survey Elements With Differing Responses Between ERAS and Non-ERAS Hospitals

The use of NA for CD, time to ambulation, time to first PO fluids, and days to discharge varied between groups (Table 3). In the ERAS group, 46% of parturients received NA for elective CD and 36% were given NA for urgent CD over 50% of the time; only 9% of non-ERAS hospitals use NA for elective CD and 6% for urgent CD >50% of the time (*P* < 0.01 for both comparisons). PO fluid intake was allowed in 91% of patients in the ERAS group within 12 h of delivery, compared to 31% of the non-ERAS group (*P* < 0.01). Thirty-six percentage of ERAS group patients were discharged within 3 days of delivery, vs. none in the non-ERAS group, and no patient stayed after 6 days in the ERAS group compared to 20% in the non-ERAS group (*P* < 0.01). Hospitals in the ERAS group had significantly more deliveries and more patients walked on the day of their CD in the non-ERAS vs. the ERAS group.

**Table 3:** Survey items with differing responses from institutions with and without ERAS protocols.

Item	Overall	ERAS protocol	No ERAS protocol	*P* value
1. NA for scheduled CD				<0.01
<10%	17 (39%)	1 (9%)	16 (49%)	
10–29%	13 (29%)	2 (18%)	11 (33%)	
30–49%	6 (14%)	3 (27%)	3 (9%)	
>50%	8 (18%)	5 (46%)	3 (9%)	
2. NA for urgent CD				<0.01
<10%	28 (62%)	2 (18%)	26 (77%)	
10–29%	8 (18%)	3 (27%)	5 (14%)	
30–49%	3 (7%)	2 (18%)	1 (3%)	
>50%	6 (13%)	4 (36%)	2 (6%)	
3. First ambulation				0.04
Day of CD	24 (53%)	3 (27%)	21 (62%)	
First post-operative day	20 (44%)	7 (64%)	13 (38%)	
Second post-operative day	1 (2%)	1 (9%)	0	
4. First PO fluids				<0.01
Immediately after CD	6 (13%)	2 (18%)	4 (11%)	
12 h after CD	15 (33%)	8 (73%)	7 (20%)	
24 h after CD	23 (50%)	1 (9%)	22 (63%)	
48 h after CD	2 (4%)	0	2 (6%)	
5. Number of deliveries				0.04
<500	28 (61%)	5 (46%)	23 (66%)	
501–1,000	12 (26%)	2 (18%)	10 (28%)	
>1,000	6 (13%)	4 (36%)	2 (6%)	
6. Days to discharge after CD				<0.01
<3 days	4 (9%)	4 (36%)	0	
3–6 days	35 (76%)	7 (64%)	28 (80%)	
>6 days	7 (15%)	0	7 (20%)	

*P-values calculated using the Pearson Chi square test*.

*CD, cesarean delivery; NA, neuraxial anesthesia; h, hours; ERAS, enhanced recovery after surgery*.

## Discussion

This is the first survey of ERAS use in obstetrics in a middle-income country. In Serbian hospitals, patients are not encouraged to eat solid food early after surgery, early mobilization is not encouraged, and a bowel preparation is mandatory in almost all hospitals. There is also a low percentage of NA for CD and a low rate of neuraxial narcotics use.

The low rate of skin-to-skin contact during CD may be due to a low rate of NA use and may interfere with successful breast feeding. Lack of successful breastfeeding is among those factors that have been associated with longer lengths of stay after CD in the UK (16).

A quarter of hospitals in Serbia reported having adopted ERAS protocols. A similar type survey conducted in the UK by Aluri and Wrench, showed that only 10 of 158 labor units in the UK were specifically following one (22). Despite this larger reported use, many elements in the ERAS protocols implemented elsewhere have not been routinely adopted by hospitals in Serbia. Most hospitals, both those with ERAS and those without ERAS, admit patients the night before elective CD, use a bowel preparation, do not allow oral intake up until a few hours before the procedure, do not routinely remove urinary catheters on the day of operation, do not monitor perioperative maternal temperature, and do not engage in active measures to avoid maternal hypothermia. These are practices that differ from hospitals outside Serbia that report using ERAS protocols. Although use of maternal temperature monitoring is routine in less than half of labor and delivery units in the UK, use of the other ERAS elements appears widespread among patients who undergo CD (21, 22).

In 2012, Abel et al. reported on a 2-month use of ERAS protocols for CD and compared two groups of 60 parturients who followed ERAS and non-ERAS pathways (19). Drinking in the recovery room was encouraged and patients were fed and mobilized early. The length of stay (LOS) decreased from 3.3 to 2.1 days, and the readmission rate was reduced from 8.3% to 3.3%. They showed that most of the patients in the ERAS group (97.8%) were satisfied and would recommend ERAS to others undergoing CD or would choose it for themselves in the future. We do not know if patient acceptance would be similar in Serbian mothers as we did not include measures of patient satisfaction in our survey.

Wrench et al. reported initiation of an ERAS for elective CD in 2012 and noted a decrease in LOS and patient satisfaction. Over 2 years, the maternal discharge rate on Day 1 increased from 1.6 to 25.2% (16). In Serbian hospitals, mothers are almost never discharged after CD on postoperative days 1 or 2, although many mothers express the desire to do so. Wrench et al. reported initiation of an ERAS for elective CD in 2012 and noted a decrease in LOS and patient satisfaction. Over 2 years, the maternal discharge rate on Day 1 increased from 1.6 to 25.2% (16). In Serbian hospitals, mothers are almost never discharged after CD on postoperative days 1 or 2, although many mothers express the desire to do so. One reason may be that the cost to the patient of in hospital care is low and patients may not feel an economic incentive to leave. Hospital administrators may also have little economic incentive to do so either. Finally, robust follow-up after patients are discharged may be necessary for patients discharged sooner after CD and the out of facility Serbian health-care system may not be able to assist parturients who are discharged home early.

We showed that Serbian hospitals that report ERAS use discharge patients home earlier than in non-ERAS groups. However, many ERAS hospitals reported a high number of discharges after 3 days compared to surveys of UK hospitals that show that a majority of women are eager go home and are discharged within 2 days of their CD. The elements of an ERAS program that are most associated with a greater chance for early discharge have not been determined. Although we showed that few elements of ERAS protocols reported elsewhere are routinely used in Serbian hospitals, adoption of an ERAS protocol lead to earlier patient discharges. We suggest that the adoption of an ERAS protocol leads to a greater effort on part of hospital staff and patients to achieve earlier maternal discharge, independent of the elements within the protocol. This change in organization and the re-setting of both staff and patient expectations may be the most important reason for reducing hospital stay, as has been suggested by others (12).

The lower rate of early ambulation in the ERAS group vs. the non-ERAS group may reflect the greater use of NA in the ERAS group. Practitioners in Serbia may discourage ambulation in patients for fear of post dural puncture headache (PDPH) after NA, despite substantial work that suggests that the rate of PDPH after NA is not influenced by patient activity.

The cost benefits of ERAS adoption for CD may be considerable. Pilkington et al. (16) reported a possible reduction of 200,000 euros in hospital expenses after implementation of ERAS protocol for CD in their hospital.

All patients may not be suitable candidates for ERAS. The factors suggesting which patients would benefit most from ERAS protocols have not been determined. However, Lucas and Gough suggested that a successful ERAS implementation for CD requires at a minimum good patient education, good in-hospital care, and good community out-of-hospital care for early discharged mothers and babies. Coates et al. showed how implementation of an ERAS program increased maternal satisfaction, primarily by increasing the number of earlier discharges (22). They also noted that a significant improvement in neonatal assessment and early determination of a neonate’s status was essential for safe early discharge of both mother and child.

Our survey study has many limitations. As noted earlier, we did not survey for patient satisfaction, which would help in deciding if ERAS protocol introduction would be appropriate. We asked either the Chief of Obstetrics or Chief of Anesthesiology to complete the survey and there may be differences between groups of respondents, although follow-up to ensure understanding of the elements of the survey tool by author BP was rigorous and should have removed this bias. We did not ask the respondents about what barriers prevent the adoption of ERAS protocols, and thus using our data to affect change only identifies those elements for improvement, not how best to implement them. We asked many questions to help to describe perioperative CD care. This may have lead to our finding differences between ERAS and non-ERAS groups that may not be real. Finally, the elements of ERAS protocols which lead to improved maternal and neonatal outcomes have not been determined.

We found that hospitals that have adopted ERAS protocols discharge patients earlier than those that have not, although they do not use many of the elements reported from the UK. We simply do not know which elements are the most important. Patient education and institutional culture change may be the vital change needed. We suggest that setting the patient’s expectation that they would be discharged home early and setting staff expectations for the same may be more important than other factors in determining time to discharge after CD; we cannot determine this from our study.

## Conclusion

Enhanced recovery after surgery protocols are in use at some Serbian hospitals for surgical procedures (mostly colorectal procedures), and some report the use of some elements of ERAS protocols for have CD as well. The results showed uncommon use of antibiotic prophylaxis prior to skin incision but routine use of DVT prophylaxis, earlier oral intake, and earlier discharge from the hospital post CD compared to hospitals not reporting ERAS use. Successful ERAS protocol implementation for CD in Serbian hospitals will require the great efforts of a multidisciplinary medical staff team and the outside community.

## Author Contributions

BP, MK, MS, YS, and CB all made substantial contributions to the conceptual design of the work, in acquiring the data, and in its analysis. All helped draft the work and revised it critically. All the authors approved this copy for publication. All agreed to be accountable for all aspects of the work in ensuring its accuracy and integrity.

## Conflict of Interest Statement

The authors declare that the research was conducted in the absence of any commercial or financial relationships that could be construed as a potential conflict of interest.
